# Progress, challenges, and responsibilities in retrovirology

**DOI:** 10.1186/1742-4690-2-1

**Published:** 2005-01-11

**Authors:** Kuan-Teh Jeang

**Affiliations:** 1 the National Institutes of Health, Bethesda, MD, USA

## Abstract

In this editorial, *Retrovirology's *choice for best basic science "retrovirus paper of the year" and a perspective on challenges and responsibilities facing HIV-1 and HTLV-I research are presented.

## Progress

The beginning of a year provides an occasion to look back upon progress made over the past 52 weeks. With the end of 2004, *Retrovirology *concluded its first calendar year of publishing. In actual fact, *Retrovirology *launched as an Open Access journal the final week of February 2004 and has been publishing continuously for a little more than 10 months. Over that period, with the wonderful efforts from my 6 very capable Associate Editors (Monsef Benkirane, Ben Berkhout, Masa Fujii, Mike Lairmore, Andrew Lever, and Mark Wainberg), the journal has thrived.

The goal that we set for *Retrovirology *is to provide a visible forum for retrovirologists so that their works can be read by all in a free and openly accessible manner. What this means is that if you are a human immunodeficiency virus (HIV)-researcher and you had published a paper in *Retrovirology*, a graduate student in Sri Lanka updating his/her research protocol, an AIDS activist in South Africa looking for the latest information, and even your long-lost high school sweetheart wondering what you have been doing all these years, can all find your work (i.e. through a simple *Google *or *PubMed *search) and read your most recent findings. Perhaps more relevant to the enterprise of scientific communication is that numerous academic peers in Eastern Europe, Asia, South America, Africa and elsewhere do not have funds which would permit them to read *Cell*, *Science *or *Nature*. Hence, while some can read your research in *Cell*, *Science*, or *Nature*, all colleagues, rich or poor alike, can read your *Retrovirology *paper.

Are they reading *Retrovirology*? You bet! Our monitored statistics tell us that in 2004, the most highly accessed review article [1] published in *Retrovirology *was read by over 3,500 individuals while a comparably popular original research article [2] was read more than 2,400 times. Readers also read *Retrovirology *articles with great immediacy. Thus, a recent paper by Rana and colleagues [3] appeared in *Retrovirology *on December 27^th^, 2004; and already by December 31^st^, 2004, a short 4 days later, that study had been read 389 times. Just as readers are quick to read our papers, I am equally pleased by our unmatched speed in publishing authors'works. In 2004, based on all papers we published in *Retrovirology*, the time from submission to publication averaged 40 days. From my personal experience of publishing in other virological journals, this duration is 3 to 4 times faster than our best competitors.

Different journals/magazines recognize "Molecule of the Year", "Breakthrough of the Year", or even "Person of the Year". With this editorial, *Retrovirology *will initiate the annual recognition of the best basic science "retrovirus paper of the year". The Associate Editors and I decided that in 2004, the best basic science retrovirus paper was the work from Joseph Sodroski and colleagues describing the HIV-1 restrictive property of the tripartite motif 5 (TRIM5) protein [4]. Thus, these researchers characterized in primates a restriction factor, similar to the Friend virus susceptibility factor-1 (Fv1) in mice, which counters the ability of infecting retrovirus to establish a proviral form in target cells. In coming years, I anticipate that *Retrovirology *Editors will find it fitting to recognize a *Retrovirology *paper as the "best basic science retrovirus paper" of the preceding year.

## Challenges and responsibilities

I explain to my postdoctoral fellows that challenges are those issues which you think others should solve, while responsibilities are items that you think you should tackle. As a retrovirologist depending on how you regard yourself, pressing problems are either others' challenges or your responsibilities. I study two retroviruses, HIV-1 and human T-cell leukaemia virus type 1 (HTLV-I). The start of a new year offers me a chance to review briefly my personal bias on the important research question that confronts HIV-1 and HTLV-I, respectively.

For HIV-1, the "holy grail" remains the development of an effective vaccine against the virus. As we enter 2005, mortality from AIDS is staggering. It is estimated that in 2004, 3.5 million individuals perished worldwide from AIDS; or nearly 10,000 AIDS deaths each day. We can view this number in another way. The recent tsunami in South Asia is estimated to have caused 150,000 fatalities. AIDS in 2004 is then the equivalent of 23 tsunamis. Imagine, unrelentingly tsunami-like casualties every fortnight from people dying from HIV-1! While it is laudable that the World Health Organization has a goal to treat three million HIV-1 positive individuals globally using anti-retroviral (ARV) medicine over the next five years, that approach will unlikely address the full magnitude of the AIDS problem, especially in developing nations. On the other hand, 100 million infants (even those in remote regions of the world) receive basic vaccinations each year. This fact suggests that when an AIDS vaccine does become available, that vaccine could be logistically and practically effective.

Separately, statistics from the World Cancer Report for the year 2000 show that 5.3 million men and 4.7 million women developed malignancy, and altogether 6.2 million persons died from cancer worldwide. The American Cancer Society estimates that approximately 553,000 individuals succumb to cancer in the United States each year. I see HTLV-I, the etiological agent for adult T-cell leukaemia (ATL), as perhaps the best retroviral system for retrovirologists to study human cancer. Substantive progress has indeed been made in our understanding as to how HTLV-I transforms cells in tissue culture [5]. What remains needed is the development of a good non-human primate model to investigate the genesis of adult T-cell leukaemia by the virus *in vivo*.

## Opportunities and limitations at mid-career

I first started studying viruses in the fall of 1977 at age 19 as a MD-PhD student at the Johns Hopkins school of medicine. For me personally, 2004 marked over a quarter-century of virus-research. At age 46, the unbridled youthful optimism of 19 is now tempered by realizations of physical and career limitations (i.e. some very interesting research problems are going to take longer to resolve than the remaining span of my scientific and physical endeavor). Nonetheless, I am optimistic and hopeful that, despite enormous odds, the opportunity to see a successful AIDS vaccine will come before I leave retrovirology research in 20 or some years. Regarding *Retrovirology*, I am also optimistic that I started this project at an age that provides ample time to develop this journal into a premier research forum.

Let me conclude this writing by thanking all authors, reviewers, Editorial board members, and our wonderful staff at Biomed Central who have contributed to *Retrovirology's *progress in our first year.
